# Scrutinizing human resources for health availability and distribution in Mozambique between 2016 and 2020: a subnational descriptive longitudinal study

**DOI:** 10.1186/s12960-023-00815-7

**Published:** 2023-04-21

**Authors:** Quinhas Fernandes, Orvalho Augusto, Helena Machai, James Pfeiffer, Marco Carone, Norton Pinto, Naziat Carimo, Isaías Ramiro, Stephen Gloyd, Kenneth Sherr

**Affiliations:** 1 National Directorate of Public Health, Ministry of Health, Maputo City, Mozambique; 2 Department of Global Health, University of Washington, Seattle, WA United States of America; 3 Eduardo Mondlane University, Maputo, Mozambique; 4 Directorate of Human Resources, Ministry of Health, Maputo City, Mozambique; 5 Department of Anthropology, University of Washington, Seattle, WA United States of America; 6 Department of Biostatistics, School of Public Health, University of Washington, Seattle, WA United States of America; 7 Comité para a Saúde de Moçambique, Maputo City, Mozambique; 8 Department of Industrial & Systems Engineering, University of Washington, Seattle, United States of America; 9 Department of Epidemiology, University of Washington, Seattle, United States of America

**Keywords:** Workforce density, HRH availability and distribution, Longitudinal analysis, Mozambique

## Abstract

**Introduction:**

Overall, resilient health systems build upon sufficient, qualified, well-distributed, and motivated health workers; however, this precious resource is limited in numbers to meet people’s demands, particularly in LMICs. Understanding the subnational distribution of health workers from different lens is critical to ensure quality healthcare and improving health outcomes.

**Methods:**

Using data from Health Personnel Information System, facility-level Service Availability and Readiness Assessment, and other sources, we performed a district-level longitudinal analysis to assess health workforce density and the ratio of male to female health workers between January 2016 and June 2020 across all districts in Mozambique.

**Results:**

22 011 health workers were sampled, of whom 10 405 (47.3%) were male. The average age was 35 years (SD: 9.4). Physicians (1025, 4.7%), maternal and child health nurses (4808, 21.8%), and nurses (6402, 29.1%) represented about 55% of the sample. In January 2016, the average district-level workforce density was 75.8 per 100 000 population (95% CI 65.9, 87.1), and was increasing at an annual rate of 8.0% (95% CI 6.00, 9.00) through January 2018. The annual growth rate declined to 3.0% (95% CI 2.00, 4.00) after January 2018. Two provinces, Maputo City and Maputo Province, with 268.3 (95% CI 186.10, 387.00) and 104.6 (95% CI 84.20, 130.00) health workers per 100 000 population, respectively, had the highest workforce density at baseline (2016). There were 3122 community health workers (CHW), of whom 72.8% were male, in January 2016. The average number of CHWs per 10 000 population was 1.33 (95% CI 1.11, 1.59) in 2016 and increased by 18% annually between January 2016 and January 2018. This trend reduced to 11% (95% CI 0.00, 13.00) after January 2018. The sex ratio was twice as high for all provinces in the central and northern regions relative to Maputo Province. Maputo City (OR: 0.34; 95% CI 0.32, 0.34) and Maputo Province (OR: 0.56; 95% CI 0.49, 0.65) reported the lowest sex ratio at the baseline. Encouragingly, important sex ratio improvements were observed after January 2018, particularly in the northern and central regions.

**Conclusion:**

Mozambique made substantial progress in health workers’ availability during the study period; however, with a critical slowdown after 2018. Despite the progress, meaningful shortages and distribution disparities persist.

**Supplementary Information:**

The online version contains supplementary material available at 10.1186/s12960-023-00815-7.

## Background

Human Resources for Health (HRH), an essential pillar of any health system, are frequently limited in supply to meet population health needs, particularly in low- and middle-income countries (LMICs). Despite the lack of consensus on the ideal number of HRH for primary health care (PHC), the World Health Organization (WHO) defines fewer than 2.8 physicians, nurses, and midwives per thousand population as an unequivocal sign of a critical shortage of HRH [1–3]. Globally meaningful progress has been achieved in closing the HRH gap; nevertheless, by 2016, 76 countries had fewer than one physician per thousand population [4]. Most of these were sub-Saharan African (sSA) countries [5], which had only 2–3% of the global number of physicians [6]. Lurking under this worrisome scenario, other important subnational concerns exist, including asymmetrical distribution, absenteeism, and poor quality training [7, 8].

Several factors in the literature have been associated with HRH shortages, including resource challenges to produce, hire, and retain qualified health workers [5, 9]. HRH shortages disproportionally affect LMICs as a result of chronic reduced fiscal space related to austerity policies and unsustainable debts. Furthermore, when evidence-based distribution criteria are lacking or are poorly implemented, challenges arise to equitably apportion health workers, improve motivation, and minimize internal and external brain drain [10]. Together, these factors lead to lower staff availability and acceptability, disproportional facility-level staff completeness, and ultimately the systematic inability to deliver quality care [11]. Scrutinizing how these contextual factors interact and affect HRH availability at the point of care is critical to ensuring efficient HRH management.

Routine information systems are primarily designed to monitor health service delivery rather than tracking human resources, financing, stockouts, and other planning and management indicators. Therefore, these systems fail to detect critical features of systems inefficiencies, including workforce availability and disparities [12, 13]. Frequently, routine statistics rely on aggregated data which hinders the ability to track individual health workers and study temporal dynamics related to individual movements within and between administrative areas [14]. Encouragingly, contemporary and robust analytic approaches can help understand and address HRH management complexities and generate solid evidence to guide equitable HRH distribution [13]. However, barriers to obtaining the data (when it exists) and poor data quality limit the ability to close the knowledge gap on HRH [15]. Addressing these issues is critical to improving HRH management, particularly in LMICs.

In Mozambique, public and private training institutions produce more health workers than can be absorbed. However, like many other LMICs, Mozambique continues to struggle to hire and equitably distribute adequate numbers of health workers to staff PHC services [16]. Furthermore, the private health sector is limited in availability and is concentrated in large urban areas; therefore, it is not an alternative for the vast majority of people [17]. Between 2014 and 2016, less than 50% of the budget needed to hire new health workers was allocated to the health sector [16]. Despite financial barriers, the Mozambique health system increased the overall number of health workers between 2006 and 2015 (from 69 to 100 per 100 000 people), for key PHC cadres, including physicians (from 4 to nearly 8 per 100 000 people) and midwives (from 34 to 48 per 100 000 people) [16]. While this increase is notable, progress has been insufficient to accomplish the ambitious goals defined in the Mozambique Ministry of Health Strategic Plan nor meet the WHO minimum HRH threshold. Furthermore, weak compliance with distribution criteria contributes to persistent disparities (gender and location) between and within country administrative areas [16]. For example, the absolute difference in HRH availability in urban versus rural areas was 111 per hundred thousand people in 2015 (176 per hundred thousand people in urban areas compared with 65 per hundred thousand people in rural areas). If evidence-based measures are not urgently taken, efforts to address the overall availability gap may fail, jeopardizing the achievement of national goals.

This study aimed to provide a detailed picture of HRH availability and distribution trends between 2016 and 2020. Furthermore, it provides an alternative and replicable approach to generate evidence on HRH by combining and validating available data sources and applying robust analytical methods to describe distribution trends.

## Methods

### Study design

We performed a district-level longitudinal analysis, using data generated by the Health Personnel Information System (eSIP-Saúde), to describe patterns in health workforce density (HWD) and the ratio of male to female health workers between January 2016 and June 2020 across all districts in Mozambique.

### Study setting

In 2023, Mozambique has around 32.4 million people and is divided into 11 provinces (admin level 2) and 154 districts (admin level 3), including the capital city, Maputo [18]. In 2018, Mozambique had 1643 health facilities, of which 1575 were primary-level, 54 secondary, seven tertiary, and seven quaternary—all hierarchically organized to ensure a comprehensive referral system and continuum of care. Typically, tertiary and quaternary health facilities are provincial or regional referral hospitals located in densely populated urban areas. The type II health center is the smallest health facility providing PHC services in rural areas with essential staffing that should include a clinician (basic or mid-level technician), a maternal and child health nurse (MCH-N), and a public health technician or nutritionist (Ministerial Diploma No. 127, July 31, 2002). The 1170 existing type II health centers represent 71.2% of the total health facilities.

Mozambique’s health system has multiple health worker cadres; however, not all are comparable to other countries in terms of years of training and core competencies. In this manuscript, physicians were defined as all doctors regardless of being generalist medical doctors or specialist medical doctors, according to the international classification of health workers (based on ISCO, revision 2008) [19]. Maternal and child health nurses are midwifery professionals, and they plan, manage, provide, and evaluate maternal and child health care services before, during and after pregnancy and childbirth. Table 1 summarizes core competencies and training duration for selected cadres, including for community health workers (CHWs, in the Ministry of Health known as *Agente Polivalente Elementar—APE*). It also provides the International Standard Classification of Occupations (ISCO) code for the analogy to other settings.

**Table 1:** Training duration, core and non-core competencies for health worker main cadres in the Mozambique health system

Cadre	ISCO code	Training duration	Core competences	Additional competences
Physician	2211 (generalist doctor)	6 years	Diagnose, treat and prevent illness, disease, injury, and other physical and mental impairments Supervise and evaluate the implementation of care and treatment plans	Training health workers Health promotion Leadership and management
	2212 (specialist doctor)^a^	+4–5 years	Diagnose, treat and prevent illness, disease, injury and other physical and mental impairments using specialized testing, diagnostic, medical, surgical, physical and psychiatric techniques Specialized in certain disease categories, types of patient or methods of treatment Perform medical education and research activities	Health promotion Leadership and management
MCH nurse	2222	2.5 years	Family planning, antenatal care, and postpartum care Performing institutional deliveries (basic emergency obstetric care) Integrated management of child illnesses care	Immunization Basic nutritional assessment Health promotion
Nurse	2221	2 years	Inpatient-centered monitoring and care Nursing care planning, management and leadership	Nursing education Health promotion
Nutrition technician	2265	2.5 years	Nutritional status assessment, habits and eating practices Health and nutritional education at the health facility and community levels (e.g., cooking demonstrations) Supplementation for children and women, deworming Outpatient nutritional rehabilitation (moderate and uncomplicated cases)	Health promotion
Medicine technician^b^	2269	2.5 years	Diagnosis and treatment for chronic diseases and infectious diseases Pediatric and adult HIV management Sexual and reproductive health care Nutritional status assessment and treatment (acute and chronic malnutrition)	Health promotion Leadership and management
Surgery technician	2240	4 years	Performing complicated institutional deliveries (advanced emergency obstetric care) Performing surgeries (e.g., cesarean section, appendectomy and other basic obstetric and general surgical procedures)	Health worker education Health promotion
Community health worker (APEs)	3253	6 months	Health promotion Integrated management of child illnesses care (community level package) Malaria treatment Basic nursing care (small and uncomplicated injuries) Family planning distribution (oral and injectable contraceptives)	Home-based visits

^a^Specialist medical doctor completes a 4–5 residence program after becoming a generalist doctor

^b^Medicine technician qualifies also for a 4-year training to become a surgery technician

### Data sources, processing, and quality control

Data were sourced and aggregated from the eSIP-Saúde database, which contains monthly excel-spreadsheets with individual-level data by month for all Ministry of Health personnel. The spreadsheets were made available by the Human Resources for Health Observatory. The eSIP-Saúde database has evolved over the years from including only annual provincial-level aggregated data (between 2000 and 2014) to annual individual-level data (2015) and later to monthly individual-level data (since 2016). After June 2020, when broader decentralization policies were introduced, notable data availability and completeness gaps arose because health workers were split between two institutions, the provincial health service and the provincial health directorate. Therefore, we only included data through June 2020. Between January 2016 and June 2020, individual personnel records were identified using unique identifiers. For this period, essential information was extracted, including demographics (age and sex), place of work, source of salary, and category—all by month.

In addition, we included data from the district-level community health worker database (gender, location, and starting year); the 2007 and 2017 Mozambique Population and Housing Census for yearly population forecasts [18]; the 2018 Service Availability and Readiness Assessment (SARA) survey for data on facility and district locations [20]; and the Ministry of Health’s routine health information system (SIS-MA) for data on health facility type. These data sources were merged with the eSIP-Saúde database to construct a consolidated dataset.

Data cleaning and quality control proceeded as follows. First, we developed an R-based algorithm to identify unusual health worker transitions across districts, defined as health worker movement from one district (district A) to another (district B) with a return to the later district (district A) within a period of fewer than 4 months. For these cases, we assumed that the health worker permanently stayed in district A. We chose 4 months based on our experience since transitions with returns less than this interval are highly unexpected. Second, we investigated unexpected category changes, particularly from higher to lower levels (for example, from physician to nurse) to ensure that every health worker was classified in the correct category across time. We also assessed potential misclassification between MCH-N and nurses. Third, we triangulated the SARA and eSIP-Saúde data to ensure that the place of work corresponded to the right district and province. Fourth, we cross-checked the type of health facility between SARA and SIS-MA data, using the former as the gold standard. Regular meetings were held with the eSIP-Saúde team over a 6-month period to double-check all inconsistencies and cross-validate data with provincial and district managers. The few remaining observations with inconsistent data were excluded from the analysis.

### Outcomes and explanatory factors

We assessed, at the district level, two primary outcomes: health workforce density and health worker’s sex ratio. HWD is the number of health workers per 100 000 population, and sex ratio refers to the number of male to female health workers at the district level. Health workers were defined as those practicing clinical activities, professionally active in clinical activities, and licensed to practice clinical activities in the public sector [21]. It includes physicians, MCH-N, nurses, clinicians (medicine, surgery, psychiatric, laboratory, pharmacy, and dentistry technicians), and public health technicians. We excluded all clinicians primarily hired in a non-clinical setting (ex: ministry of health offices and training institutions) or holding a leadership position that conflicts with clinical activity. CHWs were excluded from the HWD calculation since they provide a limited-service package.

Two main predictors were considered. First, infrastructure availability, defined as the number of health facilities per ten thousand people measured at the district level. Second, the type of referral hospital available in each district (primary, secondary, tertiary, or quaternary).

### Study sample

Based on the study inclusion criteria (being a clinician or public health worker, holding a leadership position without conflict with clinical activity, and being hired in a clinical setting), 34 120 health workers were identified in the eSIP-Saúde database between January 2016 and June 2020, of whom 1702 health workers were excluded since we could not clearly identify their workplace after completing the data cleaning process. An additional 90 health workers were excluded for being older than 70 years of age, as the legal working age limit is 65 years for males and 60 for females (the new regulation sets 60 years for both), with few exceptions given the HRH shortage. The final sample included 32 328 health workers.

### Statistical analysis

We conducted a district-level analysis with results presented as provincial and national averages. Initial district-specific plots were constructed to visualize the data, search for suspected outliers, investigate missingness, and guide decisions on model parameterization and sensitivity analysis strategy. Summary statistics—mean and standard deviation for continuous characteristics and absolute values and relative frequencies for categorical variables—were produced to describe baseline (January 2016) health worker characteristics.

Maputo City, the country’s capital, was considered a particular case since it is the only city with urban districts. This city hosts the country’s largest referral hospital (Maputo Central Hospital), which simultaneously is the major physicians’ training center. We excluded one urban district (Khamphumo District) from the trend analysis since it is where the country referral hospital is located and is an influential outlier.

Due to several factors, including data quality across provinces (completeness and delays to remove retired health workers), comparison based on observed densities is not conservative. Therefore, we modeled the health workforce densities adjusting for infrastructure availability, type of existing referral hospital, and calendar months. Thus, to estimate health worker availability, we computed the HWD for each district time-point (month) using the number of health workers and the district population for the year of interest, multiplied by 100 000. We performed a generalized estimation equation (GEE) linear regression model with robust standard errors, a first-order autoregressive (AR1) working correlation matrix, and clustering at the district level to model the HWD [22]. Given the high skewness to the right of the density and to allow interpretation of the association on a multiplicative scale, outcomes were log-transformed. After examining the correlation matrix between successive time periods, the working correlation structure was determined (Additional file 1: model equations). Because Mozambique follows a calendar year budgetary cycle (January to January), typically, the first quarter has fewer personnel contracts; therefore, we accounted for seasonal-time effects by adding months as dummy indicators. We added a linear-spline term to accommodate hidden deviation, allowing trends to differ before and after January 2018, to estimate trends before and after this cut-off. This cut-off was preferred as it matches the beginning of a period of acute budget restrictions—from the International Monetary Fund, bilateral, and multilateral donors—to hire new health workers because of the debt scandal. Infrastructure availability was centered at one health facility per ten thousand people to allow a meaningful intercept interpretation. We let each province have its own trajectory by adding an interaction term between time and province before and after 2018. Physicians, nurses, MCH-N, and CHWs densities were also assessed individually following a similar approach. The ratio of males to females and its trend before and post-2018 was estimated using a logistic regression model performed under GEE and accounting for clustering, autocorrelations of errors, secular trends, and existing district type of referral hospital.

We repeated the analysis using linear mixed-effect models [23, 24]. Overall, the two models provided approximate results, with the GEE being more conservative. All analyses were performed in R version 4.0.3 [25]. We used a model-based approach to estimate densities and the uncertainty (95% confidence intervals) rather than reporting the densities from the raw data for several reasons, including (i) exclusion of around 5% of the total health workers; (ii) human resources databases being prone to delays to removing deceased or retired health workers, and (iii) need to adjusting for critical variables and estimate differences between and within provinces.

## Results

### Descriptive

At the baseline (January 2016), Mozambique had 22 011 health workers who fulfilled the study criteria, of whom 10 405 (47.3%) were male. The mean age was 35 years (SD: 9.4). Physicians (1025, 4.7%), MCH-N (4808, 21.8%), and nurses (6402, 29.1%) together represented roughly 55% of the total study sample. Overall, 19 455 (88.4%) of the health workers were government paid. Furthermore, at baseline, there were 3122 CHWs, of whom 72.8% were male (Table 2).

**Table 2:** Baseline health worker characteristics in Mozambique (January 2016)

	Cabo Delgado	Gaza	Inhambane	Manica	Maputo City	Maputo Province	Nampula	Niassa	Sofala	Tete	Zambezia	Mozambique
Number of health workers	1553	1446	1721	1653	2538	1292	3386	1349	2569	1579	2925	22 011
Age [mean (SD)]	34.94 (9.39)	33.81 (9.15)	33.87 (9.37)	34.47 (9.52)	37.10 (10.68)	34.99 (9.05)	36.03 (9.56)	36.36 (9.46)	34.56 (10.07)	35.22 (9.61)	35.00 (9.68)	35.24 (9.73)
Age category [*N*, (%)]												
< 30 years	525 (33.8)	554 (38.3)	694 (40.3)	560 (33.9)	721 (28.4)	408 (31.6)	902 (26.6)	343 (25.4)	977 (38.0)	530 (33.6)	942 (32.2)	7156 (32.5)
30–50 years	848 (54.6)	737 (51.0)	845 (49.1)	925 (56.0)	1348 (53.1)	746 (57.7)	2061 (60.9)	845 (62.6)	1291 (50.3)	879 (55.7)	1624 (55.5)	12 149 (55.2)
> 50 years	179 (11.5)	155 (10.7)	182 (10.6)	168 (10.2)	469 (18.5)	138 (10.7)	422 (12.5)	161 (11.9)	301 (11.7)	169 (10.7)	359 (12.3)	2703 (12.3)
Male (%)	909 (58.5)	531 (36.7)	656 (38.1)	845 (51.1)	751 (29.6)	416 (32.2)	1765 (52.1)	796 (59.0)	1251 (48.7)	780 (49.4)	1705 (58.3)	10 405 (47.3)
Category [*N*, (%)]												
MCH nurses	351 (22.6)	304 (21.0)	467 (27.1)	363 (22.0)	449 (17.7)	305 (23.6)	796 (23.5)	330 (24.5)	532 (20.7)	357 (22.6)	554 (18.9)	4808 (21.8)
Nurses	368 (23.7)	436 (30.2)	441 (25.6)	477 (28.9)	892 (35.1)	278 (21.5)	945 (27.9)	393 (29.1)	852 (33.2)	414 (26.2)	906 (31.0)	6402 (29.1)
Physicians	45 (2.9)	53 (3.7)	73 (4.2)	58 (3.5)	304 (12.0)	75 (5.8)	122 (3.6)	44 (3.3)	112 (4.4)	48 (3.0)	91 (3.1)	1025 (4.7)
Dentists	23 (1.5)	39 (2.7)	37 (2.1)	31 (1.9)	39 (1.5)	46 (3.6)	70 (2.1)	19 (1.4)	47 (1.8)	33 (2.1)	45 (1.5)	429 (1.9)
Pharmacists	116 (7.5)	67 (4.6)	110 (6.4)	114 (6.9)	186 (7.3)	80 (6.2)	243 (7.2)	104 (7.7)	185 (7.2)	131 (8.3)	199 (6.8)	1535 (7.0)
Laboratorians	100 (6.4)	76 (5.3)	127 (7.4)	115 (7.0)	181 (7.1)	87 (6.7)	233 (6.9)	69 (5.1)	169 (6.6)	157 (9.9)	217 (7.4)	1531 (7.0)
Medical technicians	272 (17.5)	255 (17.6)	181 (10.5)	232 (14.0)	122 (4.8)	164 (12.7)	467 (13.8)	201 (14.9)	349 (13.6)	192 (12.2)	434 (14.8)	2869 (13.0)
Public health technicians	151 (9.7)	114 (7.9)	159 (9.2)	121 (7.3)	72 (2.8)	155 (12.0)	270 (8.0)	85 (6.3)	157 (6.1)	111 (7.0)	293 (10.0)	1688 (7.7)
Other technicians	127 (8.2)	102 (7.1)	126(7.3)	142 (8.6)	293 (11.5)	102 (7.9)	240 (7.1)	104 (7.7)	166 (6.5)	136 (8.6)	186 (6.4)	1724 (7.8)
Salary source [*N*, (%)]												
Government	1489 (95.9)	1332 (92.1)	1655 (96.2)	1486 (89.9)	2118 (83.5)	1025 (79.3)	2976 (87.9)	1235 (91.5)	2074 (80.7)	1216 (77.0)	2849 (97.4)	19 455 (88.4)
PROSAUDE^a^	62 (4.0)	93 (6.4)	66 (3.8)	167(10.1)	365 (14.4)	255 (19.7)	408 (12.0)	114 (8.5)	495 (19.3)	362 (22.9)	57 (1.9)	2444 (11.1)
Others	2 (0.1)	21 (1.5)	0 (0.0)	0 (0.0)	55 (2.2)	12 (0.9)	2 (0.1)	0 (0.0)	0 (0.0)	1 (0.1)	19 (0.6)	112 (0.5)
Number of CHW	362	232	246	270	0	158	637	322	212	213	470	3122
Male (%)	292 (80.7)	54 (23.3)	127 (51.6)	234 (86.7)	–	50.0 (31.6)	519 (81.5)	258 (80.1)	197 (92.9)	200 (93.9)	343 (73.0)	2274 (72.8)

^a^PROSAUDE: donor funds channeled through a common budget/sector wide approach

*MCH-N* maternal and child health nurse

### HRH availability and distribution

**Fig. 1:**
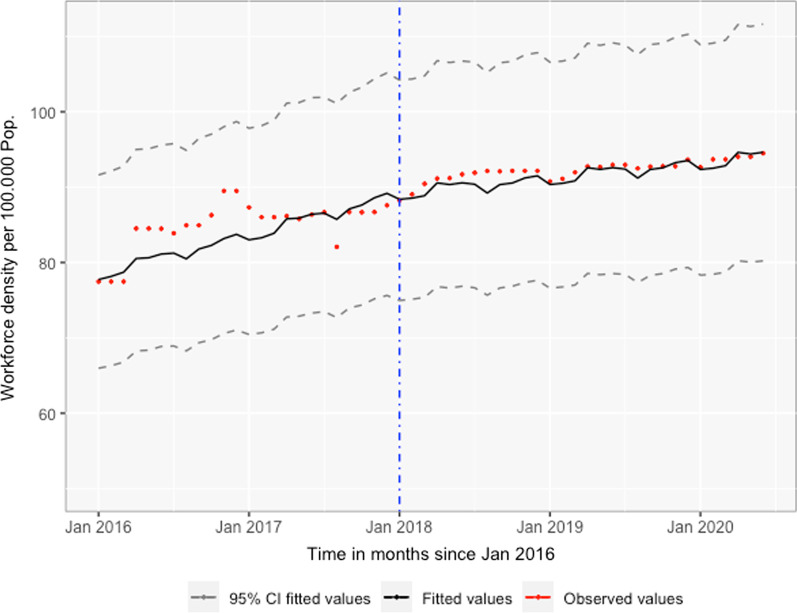
Mozambique workforce density trend from January 2016 to June 2020. The blue dotted line represents the interruption placed to assess density trends before to after 2018. The red dotted points and the black line represent the observed and the estimated from the model workforce density, respectively. The dashed dark-gray lines are the 95% confidence intervals for the estimated workforce density

**Fig. 2:**
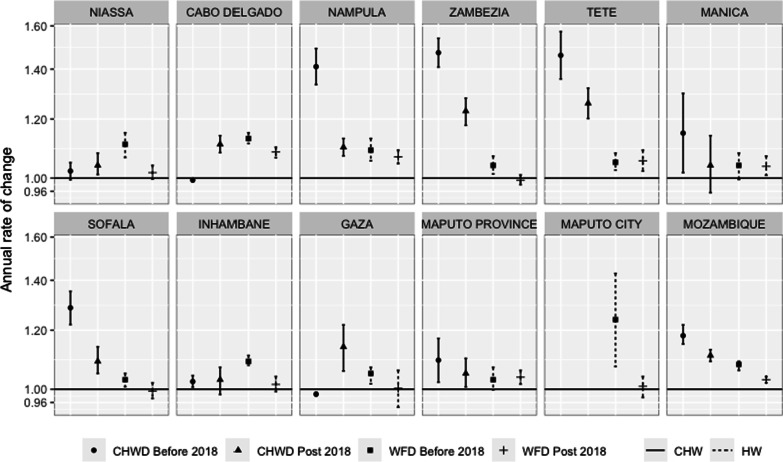
Annual rate of change of the workforce density, before and after 2018, for Community Health Workers (CHWD) and for Health Workers (HWD). There are no community health workers in Maputo City

Despite the substantial between-province variation, the average baseline district-level HWD nationwide was 75.8 per 100 000 population (95% CI 65.9, 87.1), which increased at an annual rate of 8.0% (95% CI 6.00, 9.00) through January 2018, after which it declined to an annual growth rate of 3.0% (95% CI 2.00, 4.00) (Fig. 1). On average, Maputo City, with 268.3 health workers per 100 000 population (95% CI 186.10, 387.00), and Maputo Province, with 104.6 health workers per 100 000 population (95% CI 84.20, 130.00), had the highest HWD at baseline (Table 3). Excluding Maputo City, six out of ten provinces had lower HWD compared to Maputo Province in January 2016, including Tete (45.0%), Zambézia (40.2%), Nampula (38.8%), Niassa (38.4%), Manica (31.3%), and Cabo Delgado (25.5%) (Additional file 2: Table S1). All provinces but Maputo province and Manica observed significant annual improvements between January 2016 and January 2018, ranging from 3.0% in Sofala to 24.0% in Maputo City (Fig. 2). Relative to districts with a secondary referral hospital, districts with a tertiary referral hospital had 3.9 times higher HWD (95% CI 3.13, 4.85) (Additional file 2: Table S1). Furthermore, every year, HWD was consistently higher from April to August and in the last 2 months of the year, relative to January.

**Table 3:** Overall estimated workforce density per 100 000 population in Mozambique, January 2016 and January 2018

	Estimated^a^ workforce density per 100 000 Pop. (95% CI)		Estimated^a^ physicians’ density per 100 000 Pop. (95% CI)		Estimated^a^ MCH-N density per 100 000 Pop. (95% CI)		Estimated^a^ Nurses’ density per 100 000 Pop. (95% CI)		Estimated^a^ CHW density per 100 000 Pop. (95% CI)	
	January 2016	January 2018	January 2016	January 2018	January 2016	January 2018	January 2016	January 2018	January 2016	January 2018
Mozambique	75.8 (65.90, 87.10)	88.0 (76.2, 102.0)	2.31 (1.87, 2.84)	2.5(2.03, 3.07)	51.70 (44.70, 59.80)	56.5 (48.5, 65.9)	21.8 (19.00, 25.10	25.4 (22.1, 22.1)	1.33 (1.11, 1.59)	1.86 (1.55, 2.24)
Cabo Delgado	78.0 (64.1, 94.9)	99.8 (82.5, 120.8)	2.2 (1.63, 3.01)	2.2 (1.7, 2.9)	51.0 (42.9, 60.6)	55.8 (47.7, 65.3)	18.8 (14.7, 24.0)	26.1 (20.7, 32.8)	2.0 (1.52, 2.68)	2.0 (1.5, 2.7)
Gaza	100.2 (85.0, 118.2)	109.5 (92.0, 130.3)	3.6 (2.60, 4.97)	3.9 (2.9, 5.1)	63.5 (54.6, 73.8)	68.0 (57.1, 81.0)	30.4 (24.0, 38.3)	30.6 (23.1, 40.5)	1.6 (1.16, 2.14)	1.5 (1.1, 2.1)
Inhambane	98.7 (83.9, 116.0)	117.8 (100.7, 137.8)	3.8 (3.03, 4.87)	3.6 (2.8, 4.7)	80.5 (67.9, 95.5)	94.9 (80.6, 111.6)	25.2 (21.1, 30.2)	29.0 (24.0, 34.9)	1.3 (1.05, 1.72)	1.4 (1.1, 1.8)
Manica	71.8 (58.1, 88.8)	77.3 (62.3, 95.9)	1.8 (1.42, 2.34)	1.9 (1.5, 2.5)	47.7 (38.2, 59.6)	46.7 (36.5, 59.7)	21.2 (16.5, 27.1)	23.0 (17.7, 29.8)	1.7 (1.11, 2.73)	2.3 (1.5, 2.6)
Maputo City^b^	268.3 (186.1, 387.0)	411.4 (256.5, 659.9)	25.9 (14.72, 45.67)	41.0 (24.1, 69.8)	197.6 (132.3, 295.1)	193.3 (123.9, 301.8)	58.8 (34.1, 101.6)	113.3 (55.1, 233.0)	–	–
Maputo Province	104.6 (84.2, 130.0)	111.9 (87.2, 143.6)	3.9 (2.96, 5.00)	4.8 (3.9, 5.9)	76.5 (58.0, 100.9)	79.8 (59.1, 107.7)	24.5 (19.6, 30.6)	25.4 (20.3, 31.9)	1.4 (1.03, 2.02)	1.7 (1.2, 2.5)
Nampula	64.0 (50.4, 81.0)	76.2 (61.0, 95.1)	1.9 (1.29, 2.68)	2.1 (1.5, 2.9)	43.4 (36.0, 52.4)	48.8 (40.3, 59.2)	18.7 (14.4, 24.2)	22.3 (17.8, 28.0)	1.6 (1.22, 2.22)	3.3 (2.4, 4.5)
Niassa	64.5 (53.6, 77.5)	78.7 (64.1, 96.7)	2.0 (1.35, 2.93)	2.3 (1.7, 3.0)	47.3 (40.3, 55.4)	56.6 (46.4, 59.0)	20.7 (17.0, 25.3)	23.4 (18.7, 29.2)	1.9 (1.46, 2.56)	2.0 (1.5, 2.6)
Sofala	96.2 (83.8, 110.5)	102.2 (89.9, 116.1)	2.8 (2.15, 3.54	2.9 (2.2, 3.7)	62.0 (52.7, 73.0)	63.5 (56.1, 71.9)	30.2 (26.0, 35.2)	31.8 (26.7, 37.9)	1.1 (0.81, 1.45)	1.8 (1.4, 2.3)
Tete	57.5 (46.9, 70.6)	63.9 (50.7, 80.7)	1.6 (1.15, 2.25)	1.7 (1.1, 2.5)	37.6 (31.3, 45.2)	39.7 (31.8, 49.7)	16.2 (12.7, 20.7)	18.5 (14.1, 24.2)	0.9 (0.63, 1.37)	2.0 (1.3, 3.0)
Zambézia	62.6 (51.7, 75.7)	67.7 (55.2, 83.1)	1.8 (1.36, 2.31)	2.1 (1.5, 2.9)	36.6 (30.7, 43.8)	40.6 (33.2, 49.7)	19.8 (16.2, 24.3)	21.5 (17.3, 26.7)	1.0 (0.69, 1.34)	2.1 (1.5, 2.9)

^a^The densities were estimated from a GEE model after adjusting for infrastructure availability, type of existing referral hospital and calendar months

^b^Maputo City intercept includes the coefficient for general hospitals (corresponds to a district hospital in this setting)

The disaggregated analysis demonstrates that Zambézia, Tete, Manica, Nampula, and Niassa had lower baseline densities in essential health worker categories, including physicians, MCH-N, and nurses. For example, Tete, a chronically underprivileged province, had the lowest MCH-N density at baseline (37.7; 95% CI 31.30, 45.20) and had a null slope from 2016 to 2018. Similarly, Zambézia, the second most populated province, had 36.6 (95% CI 30.70, 43.80) MCH-N per 100 000 population in January 2016, which had been increasing at an annual rate of 5.0% (95% CI 0.40, 10.00) from 2016 to 2018; however, this positive trend did not continue after 2018 (Table 4).

**Table 4:** Workforce density annual rate of change in Mozambique, before and after 2018

	Physicians’ density annual rate of change (95% CI)		MCH-N density annual rate of change (95% CI)		Nurses’ density annual rate of change (95% CI)	
	Before 2018	After 2018	Before 2018	After 2018	Before 2018	After 2018
Mozambique	1.04 (0.99, 1.08)	1.07 (1.05, 1.11)	1.05 (1.03, 1.06)	1.04 (1.02, 1.05)	1.08 (1.06, 1.10	1.02 (1.00, 1.04
Cabo Delgado	0.99 (0.91, 1.10)	1.07 (1.00, 1.16)	1.05 (1.01, 1.08)	1.14 (1.09, 1.18)	1.18 (1.14, 1.22)	1.10 (1.06, 1.13)
Gaza	1.03 (0.92, 1.16)	0.95 (0.82, 1.10)	1.04 (1.00, 1.08)	1.03 (0.99, 1.07)	1.00 (0.97, 1.04)	0.90 (0.80, 1.02)
Inhambane	0.97 (0.86, 1.09)	1.01 (1.00, 1.20)	1.09 (1.05, 1.12)	1.00 (0.98, 1.02)	1.07 (1.05, 1.10)	1.02 (0.98, 1.06)
Manica	1.02 (0.91, 1.16)	1.15 (1.05, 1.26)	0.99 (0.93, 1.05)	1.01 (0.98, 1.05)	1.04 (1.00, 1.09)	1.06 (1.02, 1.11)
Maputo City^a^	1.26 (1.10, 1.44)	1.06 (0.98, 1.13)	0.99 (0.85, 1.15)	0.97 (0.93, 1.01)	1.39 (1.04, 1.85)	1.01 (0.98, 1.05)
Maputo Province	1.12 (0.98, 1.28)	1.12 (1.02, 1.24)	1.02 (0.97, 1.08)	1.04 (1.02, 1.07)	1.02 (0.97, 1.07)	1.04 (1.02, 1.06)
Nampula	1.05 (0.90, 1.22)	1.13 (1.05, 1.23)	1.06 (1.01, 1.11)	1.08 (1.05, 1.12)	1.09 (1.05, 1.13)	1.06 (1.03, 1.09)
Niassa	1.07 (0.92, 1.23)	1.05 (0.97, 1.14)	1.09 (1.02, 1.17)	0.99 (0.96, 1.01)	1.06 (1.02, 1.11)	1.03 (0.99, 1.07)
Sofala	1.02 (0.90, 1.16)	1.04 (0.92, 1.17)	1.01 (0.98, 1.04)	1.01 (0.98, 1.03)	1.03 (0.99, 1.06)	0.97 (0.94, 1.00)
Tete	1.0 (0.91, 1.16)	1.11 (0.99, 1.24)	1.03 (0.98, 1.08)	1.07 (1.02, 1.12)	1.07 (1.03, 1.11)	1.03 (1.00, 1.06)
Zambézia	1.09 (0.97, 1.22)	0.99 (0.95, 1.05)	1.05 (1.00, 1.10)	0.99 (0.97, 1.01)	1.04 (1.01, 1.08)	0.96 (0.94, 0.98)

The annual rate of change was estimated from a GEE model after adjusting for infrastructure availability, type of existing referral hospital and calendar months

^a^Maputo City intercept includes the coefficient for general hospitals (corresponds to a district hospital in this setting)

The disproportional distribution was also evident among physicians. Tete and Zambézia provinces had 58.1% (95% CI 38.20, 71.70) and 53.9% (95% CI 36.10, 66.80) lower physician density relative to Maputo province (Additional file 2: Table S3). Although Maputo Province had the second highest increase in the annual rate of change prior to January 2018 (1.12; 95% CI 0.98, 1.28), this rate maintained and became significant after 2018 (1.12; 95% CI 1.01, 1.24) (Table 4). Not surprisingly, districts with a tertiary referral hospital observed 8.2 times higher physician density than those with a secondary hospital (95% CI 6.60, 10.23) (Additional file 2: Table S3). All provinces experienced positive changes in the density of nurses before 2018. Nevertheless, after 2018, a substantial reduction in the rate of change was observed in most provinces, with Gaza (0.9; 95% CI 0.80, 1.02) and Zambézia (4.0%; 95% CI 2.00, 6.00) reporting important reductions (Table 4).

The average number of CHWs per 10 000 population in Mozambique was 1.33 (95% CI 1.11, 1.59) in 2016 and increased by 18% annually between January 2016 to January 2018. The baseline CHW density ranged from 0.93/10 000 population in Tete to 2.02/10 000 population in Cabo Delgado. Tete and Zambézia, the two provinces with the lowest baseline CHW density, had the most notable annual increase between January 2016 and January 2018, estimated at 46.1% (95% CI 35.80, 57.20) and 47.3% (95% CI 49.90, 54.00), respectively, leading to an equitable distribution across provinces in January 2018 (Fig. 2 and Table 3).

### HRH sex ratio (male to female)

Substantial gender imbalances were observed in staffing between provinces located in the southern region compared with the rest of the country. Overall, Maputo City (OR: 0.34; 95% CI 0.32, 0.34), Maputo Province (OR: 0.56; 95% CI 0.49, 0.65), Gaza (OR: 0.71; 95% CI 0.58, 0.87), and Inhambane (OR: 0.67; 95% CI 0.58, 0.78) had significantly lower sex ratios at the baseline (Table 5). The sex ratio is more than twice as high for all provinces in the central and northern regions relative to Maputo Province. To illustrate, in January 2016, for every 100 female health workers, there were 56 males in Maputo Province [sex ratios of 0.56 (95% CI 0.45, 0.65)] while in Niassa, there were 173 [sex ratios of 1.73 (95% CI 1.47, 2.05)] (Additional file 2: Table S4 and Fig. S2).

**Table 5:** Annual rate of change (sex ratio) and rate of change in Mozambique, January 2016 and January 2018

	Adjusted odds of the sex ratio		Annual rate of change of the sex ratio (95% CI)	
	January 2016	January 2018	2016 to 2018	After 2018
Cabo Delgado	1.63 (1.46, 1.81)	1.66 (1.47, 1.88)	1.01 (0.98, 1.04)	0.94 (0.91, 0.97)
Gaza	0.71 (0.58, 0.87)	0.72 (0.59, 0.87)	1.01 (0.96, 1.06)	0.97 (0.94, 1.00)
Inhambane	0.67 (0.58, 0.78)	0.64 (0.53, 0.78)	0.98 (0.94, 1.01	0.99 (0.97, 1.02)
Manica	1.30 (1.12, 1.49)	1.18 (0.99, 1.40)	0.95 (0.92, 0.98)	0.94 (0.91, 0.99)
Maputo City	0.34 (0.32, 0.36)	0.35 (0.32, 0.39)	1.02 (0.97, 1.06)	1.00 (0.98, 1.03)
Maputo Province	0.56 (0.49, 0.65)	0.54 (0.47, 0.61)	0.98 (0.96, 1.00)	0.95 (0.91, 0.99)
Nampula	1.29 (1.16, 1.44)	1.14 (1.03, 1.26)	0.94 (0.92, 0.96)	0.95 (0.91, 0.99)
Niassa	1.73 (1.47, 2.05)	1.67 (1.41, 1.97)	0.98 (0.95, 1.01)	0.98 (0.97, 0.99)
Sofala	1.18 (1.02, 1.37)	1.22 (1.07, 1.39)	1.02 90.98, 1.05)	0.94 (0.93, 0.95)
Tete	1.16 (0.91, 1.47)	1.12 (0.89, 1.41)	0.98 (0.96, 1.01)	0.97 (0.95, 0.98)
Zambézia	1.46 (1.29, 1.66)	1.29 (1.12, 1.50)	0.94 (0.90, 0.99)	0.95 (0.93, 0.96)

Results adjusted for existing type of referral hospital

No important changes in the sex ratio were observed between 2016 and 2018, except in Manica, Nampula, and Zambezia; however, after 2018, the sex ratio continued to decrease at an annual rate of 5.0% (95% CI 1.00, 9.00) in Maputo City and Province, and 3.0% (95% CI 0.00, 6.00) in Gaza Province.

## Discussion

This study used nationally representative, granular data to describe district-level HRH availability and distribution in Mozambique. Overall, substantial improvements have been achieved in HRH availability nationwide. Despite the progress made, there was a notable slowdown in progress after 2018. The HRH availability trends after 2018 suggest a critical influence of the debt scandal since international support restrictions exacerbated the already limited fiscal space. After removing non-practicing clinicians from the analysis, the HRH gap is even more prominent than what has been officially reported. Except for Maputo City, no other province was close to the minimum HWD (2.8 per thousand people) [3, 9]. Indeed, compared to the neighboring countries, Mozambique still ranks far below the average [26].

While HRH shortages are anticipated, given current and past economic restrictions and austerity measures, critical equitable distribution issues are evident, including the following. First, the variability across provinces is unacceptably high, with some provinces having almost double the HWD compared with others. Provinces in the southern region consistently reported better HWD relative to others across time. This inequity may be due to failures in the HRH allocation criteria and/or including personnel choices, as historically, the southern region has performed better in most socio-economic indicators and is, therefore, more attractive to qualified staff [17, 18]. However, we cannot rule out a selective privilege in the allocation given to southern, particularly to Maputo City, where for example, physician density was the highest in January 2016 (more than ten times de national average) and remained with the highest increment over time. Second, gender imbalances were prominent, with the southern region predominantly staffed by females and the remaining provinces by males. Several factors could explain gender imbalances within the health sector, including poor implementation of gender equity policies in staff allocation and the countrywide implementation of public sector marriage protection policies (where a female nurse, for example, has the right to request to be hired in the same area as where her husband works) [27]. Third, even though the southern region had higher HWD levels, this did not translate into equitable distribution between districts with tertiary or quaternary-level referral hospitals and those with secondary hospitals. Indeed, this reflects another critical issue—the imbalances between the urban and rural areas—which have not been assessed directly in this study but have been described in other studies [28]. If we consider that the provincial capital cities all have a tertiary or quaternary-level referral hospital and are urbanized areas, our findings demonstrate that these areas have around four times higher HWD compared to districts with a secondary referral hospital. Therefore, the higher levels of the HWD in the southern region truly reflect the absorption of health workers in urban areas with tertiary or quaternary-level hospitals, which obscures insufficient health worker availability in rural areas [8, 29–31].

Previous studies have reported similar findings including inefficiencies in distribution criteria; disparities between production capacity and restricted absorptive capacity; weak attraction policies; poor implementation of retention policies; and internal and external migration [8, 32–34]. Nevertheless, improvements can be achieved if informed decisions and actions are taken to address inefficient HRH distribution. An example from this study is with CHWs, whose gaps have consistently decreased because the new training approach has directed resources explicitly to underprivileged provinces.

This study also demonstrates the relevance of investing in HRH databases that allow for longitudinal analysis. Although there has been a push for routine health information systems that capture outputs and outcomes such as health service provision, insufficient attention has been given to building data systems that track inputs such as human resources. Unlike aggregate monthly counts of systems outputs or outcomes, HRH databases must be designed to track individual health workers across time, requiring unique identifiers and rigid security protocols. Understanding this gap, our study leveraged a unique Ministry of Health database (eSIP-Saúde) that included a monthly census of all health workers, which was developed for record-keeping purposes to build a comprehensive database for analysis. As such, this study provides an excellent model for using existent and overlooked data sources to generate evidence to guide policymakers. As populations continue to rapidly expand in many LMICs, combined with internal migration patterns, evidence-informed decisions will be increasingly important to guide allocation and retention practices and ultimately ensure that human resources—the backbone of health systems—are in place to meet the societal health needs. Therefore, our analytic approach provides a crucial model for evidence generation.

This study has some limitations. First, we relied on routine data subject to human errors. However, our quality assessment procedures greatly improved data accuracy and reliability. Second, we excluded approximately 5% of health workers in the dataset due to the inability to clarify working locations, which could have introduced some degree of selection bias. However, the small number and the sensitivity analysis performed minimized this concern. Third, we failed to include some important factors in the model, including disease burden, development index, and health facility catchment population, leading to insufficient control of confounding. Despite these limitations, our analytic approach was robust, and results were stable in all models, enhancing our confidence in the reliability of our results.

## Conclusion

Despite the improvements in HRH availability in Mozambique, we demonstrated persistent subnational chronic disparities and gender imbalances. Importantly, our findings demand urgent changes, including (1) increases in fiscal space to allow hiring additional health workers, particularly in areas underserved; (2) updates in the distribution criteria to reduce inequalities on HRH availability and gender balance; (3) appropriate incentives and support to motivate health workers to work in rural areas; and (4) implementation of new analytic approaches to effectively track HRH trends at the subnational level and study health worker’s movements within and between administrative areas. Furthermore, this study provides a replicable model to maximize existing routine data to conduct robust analysis and provide policymakers with the information needed to drive evidence-based decisions.

## Supplementary Information

**Additional file 1.** Model equations.

**Additional file 2: Table S1.** Output from GEE model (overall health worker density). **Table S2.** Output from GEE model (maternal and child health nurse density). **Table S3.** Output from GEE model (physician density). **Table S4.** Output from GEE model (CHW density). **Table S5.** Output from GEE model (sex ratio). **Figure S1.** Province specific workforce density trend January 2016 to June 2020). **Figure S2.** Sex ratio in January 2016 (results adjusted for existing type of referral hospital). **Figure S3.** model stability for HR density. Comparing results (exponentiated betas) from GEE and linear mixed models. **Figure S4.** Model stability for HR density (comparing betas from GEE and linear mixed models).

## Data Availability

Data are available upon request to the Ministry of Health Mozambique.

## Abbreviations

CHW: Community health worker
GEE: Generalized estimation equation
HRH: Human Resources for Health
HWD: Heath workforce density
MCH-N: Maternal and child health nurses
LMIC: Low- and middle-income countries
PHC: Primary health care
SARA: Service Availability and Readiness Assessment Survey
WHO: World Health Organization
