# Supplementary material for: Scrutinizing human resources for health availability and distribution in Mozambique between 2016 and 2020: a subnational descriptive longitudinal study
Source: Hum Resour Health. 2023 Apr 21;21:33. doi: 10.1186/s12960-023-00815-7 (PMC10122375; doi:10.1186/s12960-023-00815-7)
Supplement: Supplementary file 1 — Additional file 1. Model equations. [file 12960_2023_815_MOESM1_ESM.docx]

**Model Equations**

**Equation 1: HR Density**

$$\begin{matrix} log\left( count_{dt} \right)=\beta_{0}+\beta_{1}time\_sinceJan{2016}_{t}+\beta_{2}time\_sinceJan{2018}_{t} \\ +\beta_{3}HF\_type.CS_{d}+\beta_{4}HF\_type.HP_{d}+\beta_{5}HF\_type.HC_{d}+\sum_{p=1}^{10} \gamma_{p}I\left( p=province_{d} \right) \\ +\sum_{p=1}^{10} \gamma_{10+p}I\left( p=province_{d} \right)\cdot time\_sinceJan{2016}_{t}+\sum_{p=1}^{10} \gamma_{20+p}I\left( p=province_{d} \right)\cdot time\_sinceJan{2018}_{t} \\ +\sum_{m=2}^{12} \beta_{5+m}\cdot I\left( m=month_{t} \right)+1\cdot(log\left( population_{dt}/100000 \right)+\epsilon_{dt} \end{matrix}$$

for a d district at t time (in months since 2016 January):

- count is the number of health workers
- time_sinceJan2016 and time_sinceJan2018 are the number of months since January 2016 and January 2018, respectively
- The HF terms are dummy indicators for health facility type
- p indexes the provinces in Mozambique. There are 11. One of the is a reference so we add 10 dummy indicators to the models as $\sum_{p=1}^{10} \gamma_{p}I\left( p=province \right)$
- the terms with terms including the multiplication $I\left( p=province \right)\cdot time\_sinceJan2016$ are interactions between time and province dummy indicators
- the terms containing $I\left( m=month \right)$ are month dummy indicators. There are 12 months and January is used as reference so we have 11 terms.
- the $1\cdot(log\left( population_{dt}/100000 \right)$ is the offset included in the model so the outcome becomes the ratio between counts of health care workers to the 100000 inhabitants
- $\epsilon_{dt}$ is the error term and it follows a normal distribution with mean 0 and a variance to be estimated in the model.

**Equation 2: Sex Ratio**

$$\begin{matrix} log\left( \frac{count\_males_{dt}}{count\_females_{dt}} \right)=\beta_{0}+\beta_{1}time\_sinceJan{2016}_{t}+\beta_{2}time\_sinceJan{2018}_{t} \\ +\beta_{3}HF\_type.CS_{d}+\beta_{4}HF\_type.HP_{d}+\beta_{5}HF\_type.HC_{d}+\sum_{p=1}^{10} \gamma_{p}I\left( p=province_{d} \right) \\ +\sum_{p=1}^{10} \gamma_{10+p}I\left( p=province_{d} \right)\cdot time\_sinceJan{2016}_{t}+\sum_{p=1}^{10} \gamma_{20+p}I\left( p=province_{d} \right)\cdot time\_sinceJan{2018}_{t} \\ +\sum_{m=2}^{12} \beta_{6+m}\cdot I\left( m=month_{t} \right)+\epsilon_{dt} \end{matrix}$$

for a d district at t time (in months since 2016 January):

- count is the number of health workers
- time_sinceJan2016 and time_sinceJan2018 are the number of months since January 2016 and January 2018, respectively
- The HF terms are dummy indicators for health facility type
- p indexes the provinces in Mozambique. There are 11. One of the is a reference so we add 10 dummy indicators to the models as $\sum_{p=1}^{10} \gamma_{p}I\left( p=province \right)$
- the terms with terms including the multiplication $I\left( p=province \right)\cdot time\_sinceJan2016$ are interactions between time and province dummy indicators
- the terms containing $I\left( m=month \right)$ are month dummy indicators. There are 12 months and January is used as reference so we have 11 terms
- $\epsilon_{dt}$ is the error term and it follows a normal distribution with mean 0 and a variance to be estimated in the model.
