# Supplementary material for: Scrutinizing human resources for health availability and distribution in Mozambique between 2016 and 2020: a subnational descriptive longitudinal study
Source: Hum Resour Health. 2023 Apr 21;21:33. doi: 10.1186/s12960-023-00815-7 (PMC10122375; doi:10.1186/s12960-023-00815-7)
Supplement: Supplementary file 2 — Additional file 2: Table S1. Output from GEE model (overall health worker density). Table S2. Output from GEE model (maternal and child health nurse density). Table S3. Output from GEE model (physician density). Table S4. Output from GEE model (CHW density). Table S5. Output from GEE model (sex ratio). Figure S1. Province specific workforce density trend January 2016 to June 2020). Figure S2. Sex ratio in January 2016 (results adjusted for existing type of referral hospital). Figure S3. model stability for HR density. Comparing results (exponentiated betas) from GEE and linear mixed models. Figure S4. Model stability for HR density (comparing betas from GEE and linear mixed models). [file 12960_2023_815_MOESM2_ESM.docx]

**ADDITIONAL FILE 1**

Table S1: Output from GEE Model (Overall Health Worker Density)

| Variable | Estimate | lb | ub | p.value |
| --- | --- | --- | --- | --- |
| (Intercept) | 104.62 | 84.199 | 129.994 | 0.0000 |
| time | 1.003 | 1 | 1.006 | 0.0634 |
| provinceCABO DELGADO | 0.745 | 0.565 | 0.984 | 0.0379 |
| provinceGAZA | 0.958 | 0.747 | 1.228 | 0.7340 |
| provinceINHAMBANE | 0.943 | 0.736 | 1.208 | 0.6420 |
| provinceMANICA | 0.687 | 0.519 | 0.908 | 0.0085 |
| provinceMAPUTO CIDADE | 0.658 | 0.474 | 0.913 | 0.0124 |
| provinceNAMPULA | 0.612 | 0.458 | 0.819 | 0.0009 |
| provinceNIASSA | 0.616 | 0.472 | 0.804 | 0.0004 |
| provinceSOFALA | 0.92 | 0.728 | 1.162 | 0.4820 |
| provinceTETE | 0.55 | 0.418 | 0.722 | 0.0000 |
| provinceZAMBEZIA | 0.598 | 0.46 | 0.777 | 0.0001 |
| hf_typeCS | 1.053 | 0.945 | 1.174 | 0.3510 |
| hf_typeHP | 3.899 | 3.133 | 4.853 | 0.0000 |
| hf_typeHC | 4.497 | 3.725 | 5.429 | 0.0000 |
| I(ie_avail_c/10000) | 0.816 | 0.731 | 0.911 | 0.0003 |
| time.after24 | 1 | 0.996 | 1.005 | 0.8790 |
| as.factor(month)2 | 0.999 | 0.995 | 1.003 | 0.6650 |
| as.factor(month)3 | 1 | 0.996 | 1.005 | 0.9170 |
| as.factor(month)4 | 1.017 | 1.011 | 1.022 | 0.0000 |
| as.factor(month)5 | 1.012 | 1.006 | 1.017 | 0.0000 |
| as.factor(month)6 | 1.012 | 1.006 | 1.017 | 0.0001 |
| as.factor(month)7 | 1.007 | 1.001 | 1.013 | 0.0159 |
| as.factor(month)8 | 0.991 | 0.985 | 0.998 | 0.0089 |
| as.factor(month)9 | 1.001 | 0.997 | 1.006 | 0.5840 |
| as.factor(month)10 | 1.001 | 0.996 | 1.006 | 0.6630 |
| as.factor(month)11 | 1.006 | 1.001 | 1.011 | 0.0176 |
| as.factor(month)12 | 1.006 | 1.002 | 1.011 | 0.0101 |
| time:provinceCABO DELGADO | 1.008 | 1.004 | 1.011 | 0.0000 |
| time:provinceGAZA | 1.001 | 0.997 | 1.005 | 0.6380 |
| time:provinceINHAMBANE | 1.005 | 1.001 | 1.008 | 0.0045 |
| time:provinceMANICA | 1 | 0.996 | 1.005 | 0.9080 |
| time:provinceMAPUTO CIDADE | 1.015 | 1.003 | 1.028 | 0.0163 |
| time:provinceNAMPULA | 1.004 | 1 | 1.009 | 0.0320 |
| time:provinceNIASSA | 1.006 | 1.001 | 1.01 | 0.0100 |
| time:provinceSOFALA | 1 | 0.996 | 1.003 | 0.8660 |
| time:provinceTETE | 1.002 | 0.998 | 1.005 | 0.3860 |
| time:provinceZAMBEZIA | 1.001 | 0.997 | 1.004 | 0.7810 |
| provinceCABO DELGADO:time.after24 | 0.996 | 0.991 | 1.001 | 0.1100 |
| provinceGAZA:time.after24 | 0.996 | 0.989 | 1.003 | 0.2840 |
| provinceINHAMBANE:time.after24 | 0.994 | 0.989 | 0.998 | 0.0072 |
| provinceMANICA:time.after24 | 1 | 0.993 | 1.006 | 0.9200 |
| provinceMAPUTO CIDADE:time.after24 | 0.983 | 0.969 | 0.996 | 0.0131 |
| provinceNAMPULA:time.after24 | 0.998 | 0.992 | 1.003 | 0.4500 |
| provinceNIASSA:time.after24 | 0.993 | 0.988 | 0.998 | 0.0058 |
| provinceSOFALA:time.after24 | 0.997 | 0.992 | 1.002 | 0.1830 |
| provinceTETE:time.after24 | 1 | 0.994 | 1.005 | 0.9160 |
| provinceZAMBEZIA:time.after24 | 0.996 | 0.991 | 1 | 0.0782 |

Estimated Scale Parameters: 0.0902; Estimated Correlation Parameters: alpha 0.995

Table S2: Output from GEE Model (Maternal and Child Health Nurse Density)

| Variable | Estimate | lb | ub | p.value |
| --- | --- | --- | --- | --- |
| (Intercept) | 76.4900 | 57.9890 | 100.8950 | 0.0000 |
| time | 1.0020 | 0.9970 | 1.0060 | 0.4300 |
| provinceCABO DELGADO | 0.6670 | 0.4870 | 0.9140 | 0.0117 |
| provinceGAZA | 0.8300 | 0.6150 | 1.1200 | 0.2230 |
| provinceINHAMBANE | 1.0530 | 0.7690 | 1.4400 | 0.7490 |
| provinceMANICA | 0.6230 | 0.4440 | 0.8750 | 0.0063 |
| provinceMAPUTO CIDADE | 0.7270 | 0.4840 | 1.0920 | 0.1240 |
| provinceNAMPULA | 0.5680 | 0.4140 | 0.7790 | 0.0005 |
| provinceNIASSA | 0.6180 | 0.4550 | 0.8410 | 0.0022 |
| provinceSOFALA | 0.8110 | 0.5960 | 1.1040 | 0.1830 |
| provinceTETE | 0.4920 | 0.3580 | 0.6760 | 0.0000 |
| provinceZAMBEZIA | 0.4790 | 0.3500 | 0.6550 | 0.0000 |
| hf_typeCS | 1.2680 | 1.1410 | 1.4080 | 0.0000 |
| hf_typeHP | 3.5540 | 2.6620 | 4.7460 | 0.0000 |
| hf_typeHC | 4.1440 | 3.3670 | 5.0990 | 0.0000 |
| I(ie_avail_c/10000) | 0.8120 | 0.7470 | 0.8830 | 0.0000 |
| time.after24 | 1.0020 | 0.9960 | 1.0070 | 0.5560 |
| as.factor(month)2 | 0.9990 | 0.9940 | 1.0040 | 0.7040 |
| as.factor(month)3 | 1.0040 | 0.9980 | 1.0090 | 0.2180 |
| as.factor(month)4 | 1.0190 | 1.0130 | 1.0260 | 0.0000 |
| as.factor(month)5 | 1.0140 | 1.0070 | 1.0210 | 0.0000 |
| as.factor(month)6 | 1.0160 | 1.0090 | 1.0240 | 0.0000 |
| as.factor(month)7 | 1.0120 | 1.0050 | 1.0200 | 0.0015 |
| as.factor(month)8 | 0.9980 | 0.9910 | 1.0060 | 0.6830 |
| as.factor(month)9 | 1.0110 | 1.0030 | 1.0180 | 0.0040 |
| as.factor(month)10 | 1.0080 | 1.0020 | 1.0150 | 0.0094 |
| as.factor(month)11 | 1.0100 | 1.0030 | 1.0160 | 0.0022 |
| as.factor(month)12 | 1.0110 | 1.0050 | 1.0170 | 0.0004 |
| time:provinceCABO DELGADO | 1.0020 | 0.9970 | 1.0070 | 0.4590 |
| time:provinceGAZA | 1.0010 | 0.9960 | 1.0070 | 0.6960 |
| time:provinceINHAMBANE | 1.0050 | 1.0000 | 1.0100 | 0.0526 |
| time:provinceMANICA | 0.9970 | 0.9910 | 1.0040 | 0.4420 |
| time:provinceMAPUTO CIDADE | 0.9970 | 0.9840 | 1.0110 | 0.6920 |
| time:provinceNAMPULA | 1.0030 | 0.9970 | 1.0090 | 0.2950 |
| time:provinceNIASSA | 1.0060 | 0.9990 | 1.0130 | 0.1130 |
| time:provinceSOFALA | 0.9990 | 0.9940 | 1.0040 | 0.7610 |
| time:provinceTETE | 1.0010 | 0.9950 | 1.0060 | 0.8580 |
| time:provinceZAMBEZIA | 1.0030 | 0.9970 | 1.0080 | 0.3970 |
| provinceCABO DELGADO:time.after24 | 1.0050 | 0.9980 | 1.0120 | 0.1560 |
| provinceGAZA:time.after24 | 0.9980 | 0.9910 | 1.0050 | 0.5400 |
| provinceINHAMBANE:time.after24 | 0.9920 | 0.9850 | 0.9980 | 0.0120 |
| provinceMANICA:time.after24 | 1.0000 | 0.9910 | 1.0090 | 0.9470 |
| provinceMAPUTO CIDADE:time.after24 | 0.9960 | 0.9810 | 1.0120 | 0.6410 |
| provinceNAMPULA:time.after24 | 1.0000 | 0.9930 | 1.0080 | 0.9940 |
| provinceNIASSA:time.after24 | 0.9900 | 0.9820 | 0.9970 | 0.0075 |
| provinceSOFALA:time.after24 | 0.9980 | 0.9920 | 1.0040 | 0.4850 |
| provinceTETE:time.after24 | 1.0010 | 0.9930 | 1.0100 | 0.7360 |
| provinceZAMBEZIA:time.after24 | 0.993 | 0.986 | 1 | 0.0611 |

Estimated Scale Parameters: 0.22; Estimated Correlation Parameters: alpha 0.997

Table S3: Output from GEE model (Physician Density)

| Variable | Estimate | lb | ub | p.value |
| --- | --- | --- | --- | --- |
| (Intercept) | 3.8470 | 2.9630 | 4.9950 | 0.0000 |
| time | 1.0090 | 0.9980 | 1.0210 | 0.0992 |
| provinceCABO DELGADO | 0.5760 | 0.4020 | 0.8260 | 0.0027 |
| provinceGAZA | 0.9350 | 0.6440 | 1.3580 | 0.7250 |
| provinceINHAMBANE | 0.9990 | 0.7390 | 1.3500 | 0.9940 |
| provinceMANICA | 0.4730 | 0.3510 | 0.6380 | 0.0000 |
| provinceMAPUTO CIDADE | 0.8200 | 0.4880 | 1.3800 | 0.4550 |
| provinceNAMPULA | 0.4840 | 0.3320 | 0.7070 | 0.0002 |
| provinceNIASSA | 0.5180 | 0.3390 | 0.7900 | 0.0023 |
| provinceSOFALA | 0.7170 | 0.5210 | 0.9870 | 0.0416 |
| provinceTETE | 0.4190 | 0.2830 | 0.6180 | 0.0000 |
| provinceZAMBEZIA | 0.4610 | 0.3320 | 0.6390 | 0.0000 |
| hf_typeCS | 0.9470 | 0.7970 | 1.1250 | 0.5380 |
| hf_typeHP | 8.2160 | 6.5980 | 10.2320 | 0.0000 |
| hf_typeHC | 11.3410 | 8.5350 | 15.0680 | 0.0000 |
| I(ie_avail_c/10000) | 0.8200 | 0.7440 | 0.9030 | 0.0001 |
| time.after24 | 1.0000 | 0.9840 | 1.0160 | 0.9820 |
| as.factor(month)2 | 1.0040 | 0.9940 | 1.0130 | 0.4560 |
| as.factor(month)3 | 0.9980 | 0.9870 | 1.0090 | 0.7150 |
| as.factor(month)4 | 1.0120 | 0.9960 | 1.0280 | 0.1460 |
| as.factor(month)5 | 1.0120 | 0.9970 | 1.0280 | 0.1230 |
| as.factor(month)6 | 1.0170 | 1.0000 | 1.0350 | 0.0477 |
| as.factor(month)7 | 1.0050 | 0.9870 | 1.0240 | 0.5690 |
| as.factor(month)8 | 0.9760 | 0.9590 | 0.9920 | 0.0047 |
| as.factor(month)9 | 0.9730 | 0.9580 | 0.9880 | 0.0005 |
| as.factor(month)10 | 0.9740 | 0.9590 | 0.9900 | 0.0013 |
| as.factor(month)11 | 0.9800 | 0.9670 | 0.9930 | 0.0027 |
| as.factor(month)12 | 0.9840 | 0.9720 | 0.9970 | 0.0134 |
| time:provinceCABO DELGADO | 0.9910 | 0.9770 | 1.0040 | 0.1720 |
| time:provinceGAZA | 0.9930 | 0.9790 | 1.0080 | 0.3790 |
| time:provinceINHAMBANE | 0.9880 | 0.9740 | 1.0030 | 0.1170 |
| time:provinceMANICA | 0.9930 | 0.9780 | 1.0080 | 0.3340 |
| time:provinceMAPUTO CIDADE | 1.0100 | 0.9940 | 1.0260 | 0.2280 |
| time:provinceNAMPULA | 0.9950 | 0.9780 | 1.0120 | 0.5460 |
| time:provinceNIASSA | 0.9960 | 0.9800 | 1.0120 | 0.6240 |
| time:provinceSOFALA | 0.9920 | 0.9770 | 1.0080 | 0.3210 |
| time:provinceTETE | 0.9930 | 0.9780 | 1.0080 | 0.3390 |
| time:provinceZAMBEZIA | 0.9980 | 0.9830 | 1.0130 | 0.7730 |
| provinceCABO DELGADO:time.after24 | 1.0060 | 0.9860 | 1.0260 | 0.5580 |
| provinceGAZA:time.after24 | 0.9930 | 0.9690 | 1.0170 | 0.5580 |
| provinceINHAMBANE:time.after24 | 1.0100 | 0.9890 | 1.0320 | 0.3640 |
| provinceMANICA:time.after24 | 1.0090 | 0.9870 | 1.0330 | 0.4190 |
| provinceMAPUTO CIDADE:time.after24 | 0.9850 | 0.9670 | 1.0040 | 0.1240 |
| provinceNAMPULA:time.after24 | 1.0060 | 0.9830 | 1.0300 | 0.6100 |
| provinceNIASSA:time.after24 | 0.9990 | 0.9800 | 1.0180 | 0.9000 |
| provinceSOFALA:time.after24 | 1.0020 | 0.9790 | 1.0240 | 0.8910 |
| provinceTETE:time.after24 | 1.0060 | 0.9840 | 1.0290 | 0.5870 |
| provinceZAMBEZIA:time.after24 | 0.9920 | 0.9730 | 1.0130 | 0.4570 |

Estimated Scale Parameters: 0.591; Estimated Correlation Parameters: alpha 0.993

Table S4: Output from GEE model (CHW Density)

|  | Estimate | Std.err | Pr(>\|W\|) |
| --- | --- | --- | --- |
| (Intercept) | 0.3676 | 0.1717 | 0.0323 |
| time | 0.0896 | 0.0346 | 0.00954 |
| provinceCABO DELGADO | 0.3354 | 0.2003 | 0.09397 |
| provinceGAZA | 0.0875 | 0.2156 | 0.68479 |
| provinceINHAMBANE | -0.0703 | 0.1875 | 0.70757 |
| provinceMANICA | 0.1875 | 0.2762 | 0.4972 |
| provinceNAMPULA | 0.1311 | 0.1998 | 0.5118 |
| provinceNIASSA | 0.2893 | 0.2061 | 0.16039 |
| provinceSOFALA | -0.2831 | 0.2151 | 0.18801 |
| provinceTETE | -0.4428 | 0.2295 | 0.05368 |
| provinceZAMBEZIA | -0.4114 | 0.2145 | 0.05516 |
| relevel(hf_type, ref = "HD")CS | 0.4655 | 0.0936 | 6.60E-07 |
| relevel(hf_type, ref = "HD")HP | -0.6784 | 0.1392 | 1.10E-06 |
| I(ie_avail/10000) | -0.2823 | 0.0919 | 2.12E-03 |
| time.after2 | -0.0380 | 0.0320 | 0.2344 |
| time:provinceCABO DELGADO | -0.0957 | 0.0344 | 0.00544 |
| time:provinceGAZA | -0.1051 | 0.0345 | 0.00233 |
| time:provinceINHAMBANE | -0.0656 | 0.0356 | 0.06523 |
| time:provinceMANICA | 0.0496 | 0.0712 | 0.48641 |
| time:provinceNAMPULA | 0.2549 | 0.0447 | 1.20E-08 |
| time:provinceNIASSA | -0.0680 | 0.0368 | 6.48E-02 |
| time:provinceSOFALA | 0.1617 | 0.0431 | 0.00018 |
| time:provinceTETE | 0.2898 | 0.0504 | 9.20E-09 |
| time:provinceZAMBEZIA | 0.2978 | 0.0411 | 4.10E-13 |
| provinceCABO DELGADO:time.after2 | 0.1514 | 0.0347 | 1.30E-05 |
| provinceGAZA:time.after2 | 0.1808 | 0.0501 | 3.10E-04 |
| provinceINHAMBANE:time.after2 | 0.0406 | 0.0409 | 0.3201 |
| provinceMANICA:time.after2 | -0.0572 | 0.0944 | 0.54459 |
| provinceNAMPULA:time.after2 | -0.2124 | 0.0457 | 3.40E-06 |
| provinceNIASSA:time.after2 | 0.0599 | 0.0360 | 9.65E-02 |
| provinceSOFALA:time.after2 | -0.1231 | 0.0489 | 1.19E-02 |
| provinceTETE:time.after2 | -0.1092 | 0.0589 | 6.40E-02 |
| provinceZAMBEZIA:time.after2 | -0.1437 | 0.0417 | 5.70E-04 |

Table S5: Output from GEE Model (Sex Ratio)

| Variable | Estimate | lb | ub | p.value |
| --- | --- | --- | --- | --- |
| (Intercept) | 0.5600 | 0.4860 | 0.6460 | 0.0000 |
| I(time/12) | 0.9780 | 0.9560 | 1.0000 | 0.0537 |
| provinceCABO DELGADO | 2.9040 | 2.5250 | 3.3410 | 0.0000 |
| provinceGAZA | 1.2650 | 1.0150 | 1.5770 | 0.0365 |
| provinceINHAMBANE | 1.2010 | 0.9910 | 1.4540 | 0.0619 |
| provinceMANICA | 2.3140 | 1.9790 | 2.7040 | 0.0000 |
| provinceMAPUTO CIDADE | 0.9720 | 0.8600 | 1.0970 | 0.6420 |
| provinceNAMPULA | 2.3100 | 1.9800 | 2.6950 | 0.0000 |
| provinceNIASSA | 3.0930 | 2.5980 | 3.6840 | 0.0000 |
| provinceSOFALA | 2.1090 | 1.7610 | 2.5260 | 0.0000 |
| provinceTETE | 2.0640 | 1.6090 | 2.6480 | 0.0000 |
| provinceZAMBEZIA | 2.6120 | 2.2190 | 3.0740 | 0.0000 |
| hf_typeCS | 0.9860 | 0.8880 | 1.0950 | 0.7920 |
| hf_typeHP | 0.6250 | 0.5510 | 0.7090 | 0.0000 |
| hf_typeHC | 0.6130 | 0.5580 | 0.6740 | 0.0000 |
| I(time.after24/12) | 0.9660 | 0.9250 | 1.0080 | 0.1080 |
| I(time/12):provinceCABO DELGADO | 1.0330 | 0.9940 | 1.0730 | 0.0992 |
| I(time/12):provinceGAZA | 1.0290 | 0.9720 | 1.0890 | 0.3230 |
| I(time/12):provinceINHAMBANE | 0.9980 | 0.9550 | 1.0430 | 0.9320 |
| I(time/12):provinceMANICA | 0.9740 | 0.9360 | 1.0120 | 0.1780 |
| I(time/12):provinceMAPUTO CIDADE | 1.0390 | 0.9870 | 1.0930 | 0.1450 |
| I(time/12):provinceNAMPULA | 0.9600 | 0.9290 | 0.9910 | 0.0129 |
| I(time/12):provinceNIASSA | 1.0030 | 0.9670 | 1.0410 | 0.8610 |
| I(time/12):provinceSOFALA | 1.0370 | 0.9940 | 1.0830 | 0.0912 |
| I(time/12):provinceTETE | 1.0050 | 0.9720 | 1.0400 | 0.7620 |
| I(time/12):provinceZAMBEZIA | 0.9620 | 0.9120 | 1.0140 | 0.1490 |
| provinceCABO DELGADO:I(time.after24/12) | 0.9620 | 0.9010 | 1.0270 | 0.2470 |
| provinceGAZA:I(time.after24/12) | 0.9970 | 0.9210 | 1.0800 | 0.9460 |
| provinceINHAMBANE:I(time.after24/12) | 1.0580 | 0.9870 | 1.1330 | 0.1120 |
| provinceMANICA:I(time.after24/12) | 1.0260 | 0.9490 | 1.1100 | 0.5130 |
| provinceMAPUTO CIDADE:I(time.after24/12) | 1.0210 | 0.9500 | 1.0970 | 0.5790 |
| provinceNAMPULA:I(time.after24/12) | 1.0470 | 0.9900 | 1.1080 | 0.1070 |
| provinceNIASSA:I(time.after24/12) | 1.0320 | 0.9790 | 1.0880 | 0.2370 |
| provinceSOFALA:I(time.after24/12) | 0.9600 | 0.9040 | 1.0190 | 0.1770 |
| provinceTETE:I(time.after24/12) | 1.0180 | 0.9670 | 1.0720 | 0.5040 |
| provinceZAMBEZIA:I(time.after24/12) | 1.0450 | 0.9800 | 1.1140 | 0.1760 |

Estimated Scale Parameters: 0.0144; Estimated Correlation Parameters: alpha 0.937

**
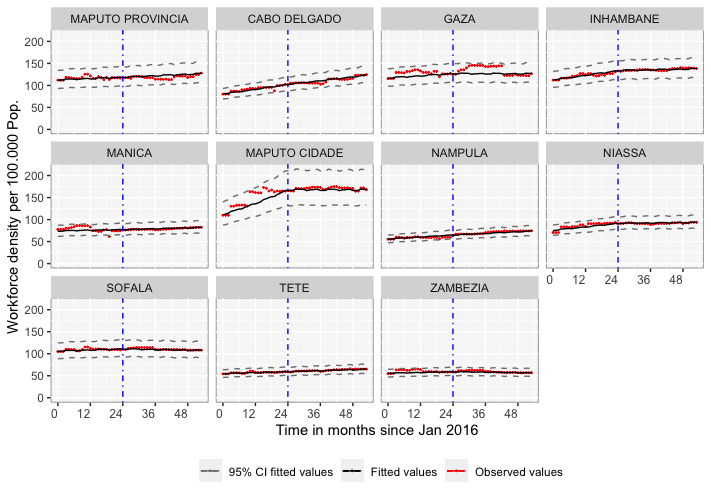
**

Figure S1: Province specific workforce density trend January 2016 to June 2020)


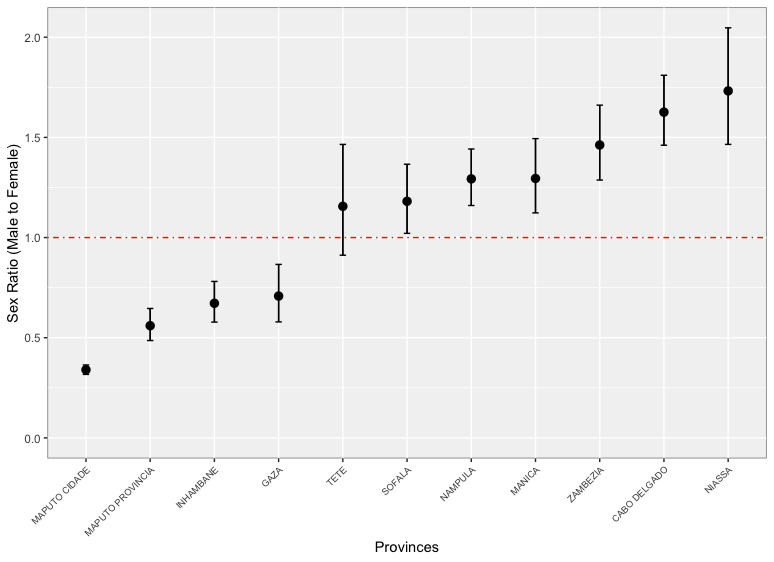


Figure S2: Sex Ratio in January 2016 (results adjusted for existing type of referral hospital)

**
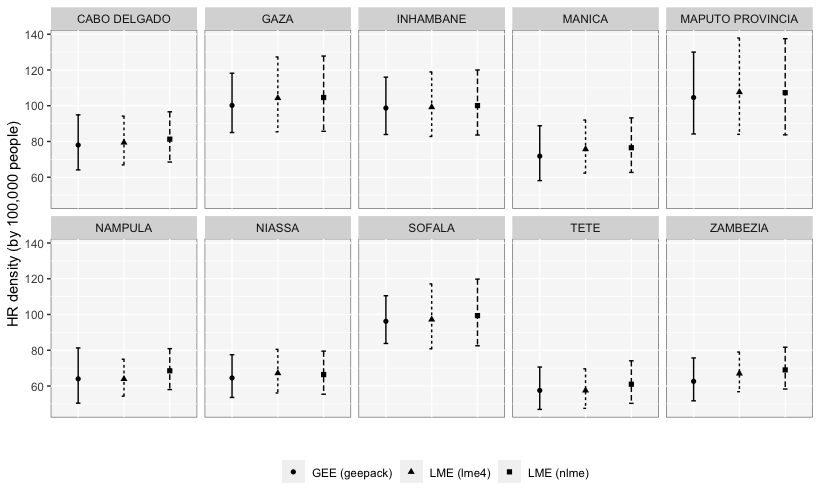
**

Figure S3: Model Stability for HR Density. Comparing Results (Exponentiated Betas) from GEE and Linear Mixed Models

**
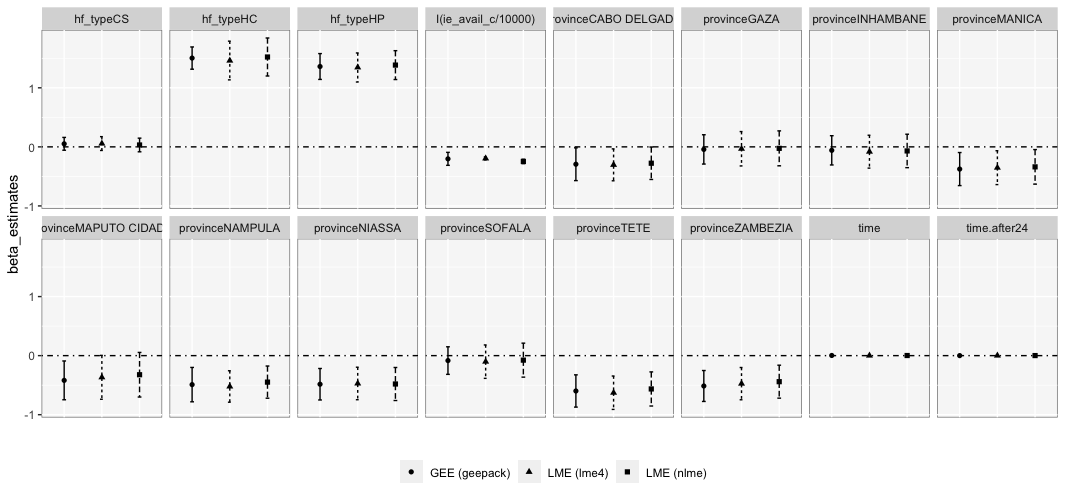
**

Figure S4: Model Stability for HR Density (comparing betas from GEE and Linear Mixed Models)
